# Editorial: New Frontiers for Artificial Intelligence in Surgical Decision Making and its Organizational Impacts

**DOI:** 10.3389/fsurg.2022.933673

**Published:** 2022-06-21

**Authors:** Lorenzo Cobianchi, Francesca Dal Mas, Luca Ansaloni

**Affiliations:** ^1^ Department of Clinical, Diagnostic and Pediatric Sciences, University of Pavia, Pavia, Italy; ^2^ Department of General Surgery, IRCCS Policlinico San Matteo Foundation, Pavia, Italy; ^3^ Department of Management, Ca’ Foscari University of Venice, Venice, Italy

**Keywords:** artificial intelligence, surgical oncology, organizational impacts, clinical decision-making, Technology

**Editorial on the Research Topic**

New Frontiers for Artificial Intelligence in Surgical Decision Making and its Organizational Impacts

Artificial Intelligence (AI) as a new technology in the Industry 4.0 scenario(1) is gaining importance in a variety of industrial and service sectors, including healthcare (2). Several algorithms were approved for clinical use worldwide, involving many clinical specialities.

As of today, AI- and Machine and Deep Learning-based applications in oncology and surgery have the primary aim of supporting clinical decision-making (3). The use of AI-based applications may be beneficial for medical doctors to guide their clinical decisions, helping them in early and more accurate diagnoses and appraising unforeseen issues. Such decision aids look particularly valuable when physicians are called to decide fast, in complex and uncertain situations and with time constraints. The current literature underlines how AI-based applications may contribute to better clinical outcomes. Still, such applications also imply the need for hospitals and institutions to understand the organizational implications, for example, in terms of investments, costs, the actual degree of efficiency and effectiveness, the relationship among the various stakeholders involved, and the educational and training plans.

The purpose of the research topic call “New Frontiers for Artificial Intelligence in Surgical Decision Making and its Organizational Impacts “ was to collect the recent developments and undergoing studies in AI in surgery and surgical oncology. More in detail, the aim was to gather contributions on the advancement, deployment, use, and implementation of AI-based applications in surgical practice, understanding their potential contribution to clinical decision making. Moreover, the idea was to assess the potential impacts of such a technology on surgeons, other clinicians, patients, medical institutions, developers, and policy-makers, with an eye open to the organizational and educational consequences and opportunities.

The special issue gathered six articles: five original research pieces and one mini-review.

In the field of neuro-oncology, the paper by Tariciotti et al. (4) reported a pilot study concerning a Deep Model algorithm trained and internally validated to reliably differentiate ambiguous cases of primary central nervous system lymphomas, glioblastoma, and brain metastasis using T1-weighted gadolinium-enhanced magnetic resonance imaging scans. The proposed predictive model, tested on 121 patients, yielded promising results, standing as a high-speed decision-making aid for diagnostic brain biopsy or maximal tumor resection in atypical cases. Organizational advantages may derive from the fact that the model is potentially low-cost and easily accessible.

Working in the field of general surgical oncology, the study by Quan et al. (5) aimed at building and validating a decision tree model to predict disease-specific survival after curative resection for primary cholangiocarcinoma. The experiment, starting from a sample of 482 patients, allowed the research group to build a sound decision tree model to foretell outcomes for cholangiocarcinoma undergoing curative resection. The model gave outstanding results than other clinical patterns, supporting the decision-making process of adjuvant therapy after curative resection.

In the same hepato-pancreato-biliary field, the study by Wu and colleagues (6) aimed to support clinicians in evaluating the hepatic functional reserve to forecast indocyanine green retention rate at 15 min to ensure safe hepatic resection. According to the study, a novel approach based on Computed Tomography images and clinical data may support clinicians in measuring the indocyanine green retention rate at 15 min in patients with hepatocellular carcinoma. The results of a retrospective study with 350 patients were promising, allowing an individualized prediction of the rate. From an organizational perspective, the model may be beneficial for institutions with fewer resources and less sophisticated pieces of equipment.

The study by Jian and colleagues (7) strived to evaluate the diagnostic performance of magnetic resonance imaging-based radiomics in the differentiation of fat-poor angiomyolipomas from other renal neoplasms. A study conducted on 69 patients allowed the research team to demonstrate how the clinical-radiomics model could represent a valuable noninvasive diagnostic tool to distinguish fat-poor angiomyolipomas from other renal neoplasms. As a result, such a model may stand as an effective decision-making aid for clinicians.

The article of Azam et al. (8) in the field of otorhinolaryngology grounds on the potentiality of AI to empower upper aero-digestive tract videoendoscopy to detect cancer and evaluate margins. A model based on convolutional neural networks is employed for automatic semantic segmentation of upper aero-digestive tract cancer on endoscopic images. A retrospective study on 219 patients with laryngeal squamous cell carcinoma allowed the research group to test the so-called “SegMENT” model. The results reached by SegMENT proved to be excellent, underlining that automatic tumor segmentation in endoscopic images is feasible even within the complex and heterogeneous upper aero-digestive tract environment. The promising model’s performance showed potential for improved tracking of early tumors, more accurate biopsies, and better selection of resection margins.

Last but not least, seeing surgery more generally, the mini-review of Balch and colleagues (9) allowed shedding light on the promising contribution of gamification, empowered by Machine Learning technology, to shared-decision making involving clinicians and patients. Advantages include limiting pre-operative complications, reducing in-hospital problems, and fostering recovery. The study leads to a theoretical mobile application able to support decision making while offering evidence-based and tangible results for all the parties involved, facilitating the optimal surgical outcomes while coping with the patient’s wishes.

The contributions to the research topic “New Frontiers for Artificial Intelligence in Surgical Decision Making and its Organizational Impacts” allowed us to explore and enjoy the potential of several AI-based applications in various surgical fields, including neurosurgery, general surgery, and otorhinolaryngology, both from a clinical but also organizational perspective. All the studies, conducted with rigorous methodologies, underline the promising and sometimes outstanding outputs of such applications, resulting in sound decision aids for surgeons and oncologists, with positive organizational impacts. The mini-review also stresses the potential role of a Machine-Learning-empowered gamification mobile app to foster the dialogue between the surgeon and the patient, leading to surgical decision-making, which is equally shared among the parties in a patient-centric perspective with multiple positive outcomes.

The encouraging results reported by the collected articles depict a bright future for the development and use of AI-based applications in surgery and oncology, with positive clinical as well as organizational impacts. A multidisciplinary approach involving clinical professionals, industry leaders, healthcare institutions, and patients is recommended to fully translate the clinical and organizational potentials of such a technology.
